# Limited implementation of California’s Healthy Default Beverage law for children’s meals sold online

**DOI:** 10.1017/S1368980022000039

**Published:** 2022-07

**Authors:** Hannah R Thompson, Anna Martin, Ron Strochlic, Sonali Singh, Gail Woodward-Lopez

**Affiliations:** 1 University of California Berkeley, School of Public Health, 2121 Berkeley Way, 6120, Berkeley, CA 94720-7360, USA; 2 University of California Agriculture and Natural Resources, Nutrition Policy Institute, 1111 Franklin, Fifth Floor, Oakland, CA 94607, USA

**Keywords:** Sugar-sweetened beverages, Healthy default beverages, Fast food restaurants, Children’s meals

## Abstract

**Objective:**

To reduce children’s sugar-sweetened beverage intake, California’s Healthy-By-Default Beverage law (SB1192) mandates only unflavoured dairy/non-dairy milk or water be the default drinks with restaurant children’s meals. The objective of this study is to examine consistency with this law for meals sold through online platforms from restaurants in low-income California neighbourhoods.

**Design:**

This observational, cross-sectional study examines beverage availability, upcharges (additional cost) and presentation of beverage options consistent with SB1192 (using four increasingly restrictive criteria) within a random sample of quick-service restaurants (QSR) in Supplemental Nutrition Assistance Program Education eligible census tracts selling children’s meals online from November 2020 to April 2021.

**Setting:**

Low-income California neighbourhoods (*n* 226 census tracts).

**Participants:**

QSR that sold children’s meals online via a restaurant-specific platform, DoorDash, GrubHub and/or UberEats (*n* 631 observations from 254 QSR).

**Results:**

Seventy percent of observations offered water; 63 % offered unflavoured milk. Among all beverages, water was most likely to have an upcharge; among observations offering water (*n* 445), 41 % had an upcharge (average $0·51). Among observations offering unflavoured milk (*n* 396), 11 % had an upcharge (average $0·38). No observations upcharged for soda (regular or diet). Implementation consistency with SB1192 ranged from 40·5 % (using the least restrictive criteria) to 5·6 % (most restrictive) of observations.

**Conclusions:**

Based on observations from restaurant websites and three of the most popular online ordering platforms, most California QSR located in low-income neighbourhoods are not offering children’s meal beverages consistent with the state’s Healthy-By-Default Beverage law. As the popularity of online ordering increases, further work to ensure restaurants offering healthy default beverages with children’s meals sold online is necessary.

Rates of childhood obesity and related chronic diseases have significantly increased in recent decades, with nearly one in five US children and adolescents having obesity^(1,2)^. Sugar-sweetened beverages (SSB) are a significant causal contributor to overweight and poor health outcomes in youth^(3,4)^. Approximately 60 % of US children consume SSB on a daily basis^(5)^, accounting for 8 % of energy intake^(3)^. Further, daily SSB consumption is higher for children of colour and children from low-income families, compared with White and higher income children, likely contributing to health disparities^(6,7)^.

On average, 25 % of children’s SSB intake is consumed in restaurants^(8)^. This is likely due at least in part to the high availability of SSB in those settings; in 2019, 61 % of the top fifty restaurant chains in the US had SSB on children’s menus^(9)^. Consumption of children’s meals from quick-service restaurants (QSR) is associated with increased energetic intake^(10)^ and increased SSB intake^(11)^. Moreover, non-White and low-income youth are more likely to regularly consume QSR food compared with their White and higher income counterparts, further exacerbating existing health disparities^(12,13)^. This could be due, in part, to a higher prevalence of QSR in low income and communities of colour^(14,15)^, as well as disproportionate QSR advertising targeted at Black and Latinx youth^(16)^. Identifying interventions to decrease youth SSB consumption is necessary, and may be successful when executed in the QSR setting.

In an effort to reduce children’s SSB consumption, several states (including California, Delaware and Hawaii) and jurisdictions (including Philadelphia, PA; New York, NY; Baltimore, MD; Louisville, KY and Lafayette, CO) have enacted legislation mandating healthy beverages (i.e. water, unflavoured milk products and in some cases, 100 % fruit juice) as the default beverage options for children’s meals sold in restaurants including QSR^(17–20)^. California’s law, Senate Bill (SB) 1192^(21)^, known as the Healthy-By-Default Beverage law, is among the most stringent of existing legislation, allowing only plain or sparkling water with no added sweeteners, unflavoured milk or unflavoured, non-dairy milk product alternatives as default beverage options (only allowable beverages are automatically included or offered as part of a children’s meal, absence of specific request by the purchaser of the children’s meal for an alternative beverage). The law also requires that menus, menu boards and advertisements for children’s meals include only approved default options. In California, customers may still purchase SSB with children’s meals, but must specifically request those beverages.

Evidence from a broad range of fields suggests that consumers tend to select default options^(22–25)^, with further evidence suggesting healthy default beverages are acceptable to children and parents and can result in more nutritious choices^(26–28)^. Studies have found increased ordering of more healthful items^(17,29)^ and reduced energetic intake^(29)^ following the implementation of healthy default menus. Additional research indicates that cost also impacts beverage choice^(30)^, with in-store studies demonstrating that pricing, in combination with promotion and prompting, effectively impacts purchasing behaviour^(31)^. However, evidence regarding the impact of healthy default policies on pricing is mixed, with some studies finding price increases^(29)^ and others reporting no change in prices^(32,33)^.

A study examining adherence to California’s Healthy-By-Default Children’s Meal Beverage law when ordering in-person at QSR (either inside the restaurant or via drive through) demonstrated the proportion of menu boards listing only healthy default beverages with children’s meals increased from 9·7 % to 66·1 % after SB1192 was enacted; however, few staff verbally offered beverages consistent with the legislation, with a significant decrease from 5 % to 1 %^(18,34)^. The incomplete implementation of the law could result in an attenuation of the intended impact to reduce SSB consumption among young children.

In recent years, there has been an increasing trend in ordering meals online (either via website or phone application) for pick-up or delivery^(35)^. With the onset of the COVID-19 pandemic in March 2020, which resulted in stay-at-home and social distancing orders, online ordering has further increased in popularity^(36)^. Whether or not QSR fully implement SB1192 when offering beverages with children’s meals sold online is therefore of increasing importance, yet remains unknown.

Collaborating with under-resourced communities, including the retail food sector, to reduce SSB consumption is a goal of CalFresh Healthy Living—the Supplemental Nutrition Assistance Program Education (SNAP-Ed) in California. The California Department of Public Health implements state-wide CalFresh Healthy Living initiatives and funds local health departments in nearly every county to implement CalFresh Healthy Living. This study was conducted to inform the work of CalFresh Healthy Living and their partners regarding the need for complementary local or state action to ensure optimal implementation and effectiveness of SB1192 in achieving the objective of reducing SSB consumption by young children. Specifically, this study examines the online ordering process for QSR located in SNAP-Ed eligible census tracts to provide specifics regarding the extent and nature of SB1192 implementation on QSR-specific and third-party online ordering platforms with the aim of informing efforts to improve policy language and provide support for policy implementation.

## Methods

### Sample

For this observational, cross-sectional study, we sampled QSR sites from California’s thirteen largest QSR chains that sold children’s meals (defined as a combination of food items and a beverage, or a single food item and a beverage, sold together at a single price, primarily intended for consumption by a child^(21)^). From within each eligible chain, we randomly sampled QSR sites located in all SNAP-Ed eligible census tracts (*n* 1350) across the state using the 2019 Dun & Bradstreet California Retail Food Environment dataset^(37)^. A census tract was considered SNAP-Ed eligible if 50 % or more of households had incomes at or below 185 % of the Federal Poverty Level.

**Fig. 1:**
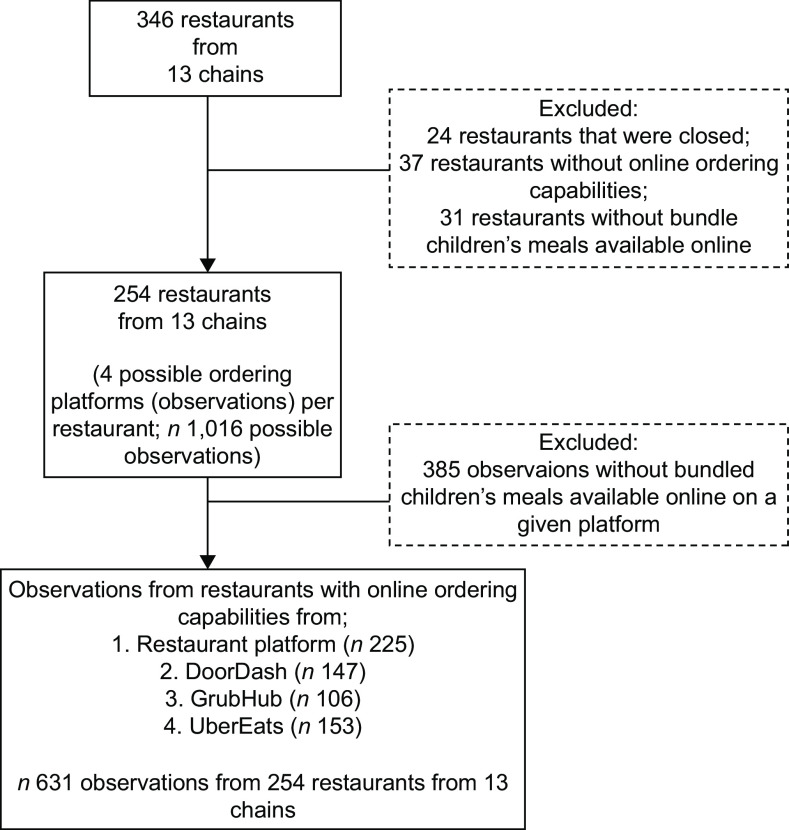
Sample restaurant and observation flow chart

For each of the QSR chains in our sample with ≥ 79 restaurants total in the study census tracts, the number of QSR sites sampled was proportional to the size of that chain relative to the other sampled chains to achieve an error of +/– 5·15 % in the estimate of whether a QSR upcharged for default beverages (water and unflavoured milk). For chains with < 79 restaurants per chain, all QSR sites in the study census tracks were sampled. This resulted in an initial sample of 346 QSR sites from 13 chains (Fig. 1). QSR sites: that were closed (*n* 24); without online ordering capabilities (*n* 37) and without children’s meals available online (*n* 31) were excluded from the sample.

From the remaining 254 QSR sites, we collected data from four potential online ordering platforms (restaurant-specific platform, DoorDash, GrubHub and UberEats) per site (*n* 1016 possible observations). Observations without children’s meals available on a given platform were excluded (*n* 385 observations). The final sample included 631 observations from 254 QSR sites from thirteen chains.

The study sample of 254 QSR sites was located in 226 California SNAP-Ed eligible census tracts (Table 1). There are on average 953 children ages 0–11 living in these census tracts, 36·1 % of whom live under 100 % of the federal poverty line and 67·6 % of whom live under 185 % of the federal poverty line. On average, census tract residents were primarily Hispanic or Latinx (62·4 %) or White (non-Hispanic) (20·9 %).

**Table 1:** Demographic characteristics of census tracts in which the sample of 254 restaurants reside, (*n* 226 census tracts)

Population	Mean	sd
All ages	5010	2106
Ages 0–11	953	553
Proportion (%) of population under 100 % of the federal poverty line		
All ages	27·8	10·1
Ages 0–11	36·1	14·7
Proportion (%) of population under 185 % of the federal poverty line		
All ages	54·5	0·91
Ages 0–11	67·6	15·0
Proportion (%) of population by race/ethnicity*		
American Indian/Alaska Native, non-Hispanic	0·5	2·2
Asian, non-Hispanic	8·4	11·3
Black, non-Hispanic	7·7	8·6
Hispanic or Latinx	62·4	23·5
White, non-Hispanic	20·9	19·1
Two or more races/other	2·9	3·0

* Rounded averages may not add up to 100 %.

### Data collection tool

Study data were collected from November 2020 to April 2021 using a standardised protocol and a data collection instrument adapted from an instrument previously used for assessing on-site restaurant menu compliance with SB1192^(20)^. Each QSR chain was assigned one pre-determined kid’s meal entrée to be ‘ordered’ during data collection for all restaurants and ordering platforms for that chain to ensure that any price differences seen within a chain were due only to differences in beverage selection. The selected entrée was usually the first entrée listed at the smallest size without an upcharge. For example, one QSR chain that offered multiple sizes and options for children’s meals entrees was assigned a hamburger as the standard entrée, and all data collected from that chain (from every restaurant and every platform) were related to a children’s meal with a hamburger.

#### Beverage availability

Beverages available with children’s meals were categorised as follows: water bottle or cup; unflavoured milk (regardless of fat content); unspecified milk (unclear if flavoured/unflavoured); flavoured milk (e.g. chocolate) regardless of fat content; 100 % fruit juice with no added sugar; juice diluted with water and no added sugar; unspecified fountain/kids drink (listed as ‘fountain drink,’ ‘kids’ drink’, ‘small drink’ or something similarly non-specific, often with a link or drop-down menu with specific beverage options, including SSB); regular soda; diet soda; soda (unclear if regular or diet); other pre-sweetened beverages (e.g. sweetened iced teas, sweetened lemonades, sweetened juice drinks) and other unsweetened or artificially sweetened beverages (unsweetened iced tea, lite lemonade). Data collectors recorded whether and which beverages were offered initially when ordering online (on the first screen where beverage selection was available) and, in cases where additional options were available, on a second screen that the customer was directed to if they selected an option to see additional beverage choices. Data collectors also recorded if and which beverages were included in images of the full kids’ meal (that included all kid’s meal components). No data were collected regarding images of only beverages that were placed next to a listed beverage option on the kids’ meal ordering screen(s).

#### Costs

If a beverage choice increased the total cost of the children’s meal (e.g. choosing milk increased the meal price by $1·00), then the additional cost (upcharge) was recorded. The total cost of the children’s meal with the specified entrée was also recorded. For each beverage offered with an upcharge, the upcharge amount was divided by the cost of the children’s meal to determine the upcharge as a proportion of the total children’s meal cost.

#### Making healthy beverages the default/Consistency with SB1192

**Fig. 2:**
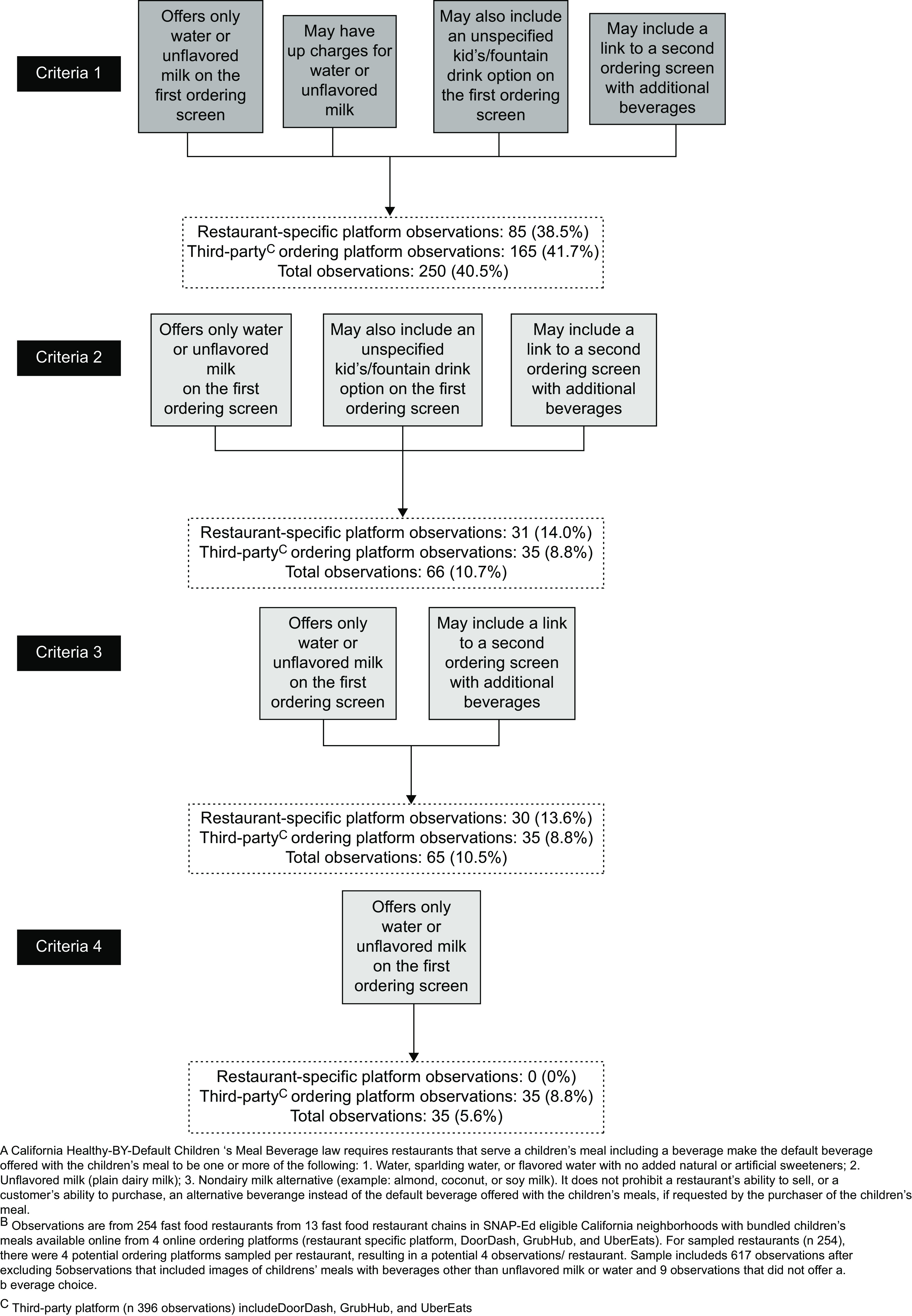
Implementation of California’s Healthy-by-Default Children’s Meal Beverage law^A^ using progressively more restrictive criteria (*n* 617 observations^B^ from four ordering platorms: restaurant-specific (*n* 221), DoorDash (*n* 145), GrubHub (*n* 106) and UberEats (*n* 145))

SB1192, as written, does not specifically mention online ordering, lacks details regarding how other (non-compliant) beverages can be offered in the online context and fails to clarify if there can be additional costs (upcharges) for default beverages. ‘Compliance’ with the law is therefore subject to interpretation, and thus difficult to assess. To address this challenge, we developed four increasingly restrictive sets of criteria to assess the extent of implementation of the law (Fig. 2). This approach also supports the study objective by providing more nuanced information to inform improvements in policy language and implementation efforts. As written, the law clearly does not allow the initial offering of specific non-compliant beverages; therefore, all four criteria specify that one or both of the allowable default beverages (water and/or unflavoured/unspecified milk) be offered and no other specifically named beverages be offered on the first children’s meal beverage ordering screen (i.e. only initial offering of default beverages). The criteria also concern upcharges and how the purchaser can ‘request’ or access other beverages, which were not written into the law, but are directly related to the law’s intent to reduce children’s SSB consumption by making the healthy choice the easiest choice.

Criteria 1, the most lenient criteria (which allows for the most flexibility in the interpretation of the law as it pertains to meals sold online) allows: (a) only initial offering of default beverages, (b) upcharges for the allowable default beverages, and allows for the two ways that sites provided access to other drink options, (c) an unspecified kids’/fountain drink option on the first beverage ordering screen usually with a link or drop-down with other drink options and (d) a link with wording such as ‘other beverages’ to a second ordering screen with additional beverages. Criteria 2 allows for only (a), (c) and (d). Criteria 3 allows for only (a) and (d). Finally, the most strict criteria, Criteria 4 only allows for (a). Because of their limited number, and because not all images (such as those associated with listed beverages options) were assessed, the five observations where images of the children’s meal included a beverage other than the allowable defaults were excluded from this assessment of implementation as were the nine observations that did not include the option to select a beverage.

### Data analysis

Beverage availability, consistency of beverage availability within the same QSR site across all platforms and average beverage upcharge data were calculated using descriptive statistics. The number and proportion of observations by platform type (restaurant-specific *v*. third-party (DoorDash, GrubHub and UberEats) and extent of implementation based on the four criteria were also calculated. All analyses were performed in Stata/MP v16 (College Station, Texas).

## Results

Most QSR sites had online ordering capabilities on a restaurant-specific platform (*n* 225; 89 % of QSR sites); followed by UberEats (*n* 153; 60 %), DoorDash (*n* 147; 58 %) and GrubHub (106; 42 %).

Seventy percent of observations offered water on either the first or second screen; 62·8 % offered unflavoured milk, 24·3 % offered unspecified milk and 51·7 % offered 100 % fruit juice (Table 2). Overall, 622 observations (99 %) had beverages available on the first beverage ordering screen. Nine observations (1 %) did not enable beverage selection (presumably, only one beverage came, pre-selected, with the children’s meal or a beverage choice was possible at meal pick-up). The most common beverages offered on the first ordering screen were water (70·4 % of observations), unflavoured milk (61·8 %) and 100 % fruit juice (39·8 %). On average, QSR were least consistent in offering water on the first screen across all platforms (only offered consistently for 44·5 % of QSR). Unflavoured milk was offered consistently on the first screen across all platforms for 61·8 % of QSR sites.

**Table 2:** Beverage availability, consistency and pricing with bundled children’s meals available to order online in fast-food restaurants in Supplemental Nutrition Assistance Program Education eligible neighbourhoods in California, (n 631 observations*, 254 restaurants, 13 restaurant chains)

	Observations that offered beverage with children’s meals on either first or second ordering screen†, (*n* 631 observations)		Observations with beverage available with children’s meal on first ordering screen, (*n* 631 observations)		Restaurants with consistent availability of beverage on first ordering screen across all platforms, (*n* 254 restaurants)		Observations with beverage shown in an image of the children’s meal‡, (*n* 631 observations)		Observations with beverage available with children’s meal on second ordering screen, (*n* 132 observations)		Restaurants with consistent availability of beverage on second ordering screen across all platforms, (*n* 105 restaurants)		Of observations that offered this beverage, number that up-charged for this beverage		Of observations that upcharged for this beverage, average price of upcharge		Of observations with an upcharge for this beverage, upcharge as average % of total children’s meal cost	
Beverage	*n*	%	*n*	%	*n*	%	*n*	%	*n*	%	*n*	%	*n*	%	$	range	%	range
Beverages allowed as defaults by California’s Healthy-by-Default Children’s Beverage law§																		
Water	445	70·5	444	70·4	113	44·5	52	8·2	86	65·2	20	19·1	183	41·1	0·51	0·10–1·29	11·1	2·0–31·7
Milk, unflavoured	396	62·8	390	61·8	157	61·8	376	59·6	78	59·1	28	26·7	42	10·6	0·38	0·04–1·10	7·4	0·8–17·3
Milk, unspecified||	153	24·3	153	24·3	150	59·1	99	15·7	0	0·0	105	100	31	20·3	0·27	0·10–0·69	5·4	2·0–17·3
Other beverages (not allowable defaults by California’s Healthy-by-Default Children’s Beverage law§)																		
Milk, flavoured	223	35·3	152	24·1	203	79·9	0	0·0	71	53·8	34	32·4	10	4·5	0·32	0·10–0·70	7·3	1·9–17·3
Fruit juice, 100 %	326	51·7	251	39·8	169	66·5	1	0·2	75	56·8	30	28·6	92	28·4	1·03	0·20–2·40	23·9	3·2–68·6
Fruit juice, diluted	166	26·3	93	14·7	246	96·9	0	0·0	73	55·3	32	30·5	13	7·8	0·43	0·20–0·90	9·5	4·2–20·1
Soda, regular	274	43·4	156	24·7	191	75·2	0	0·0	118	89·4	13	12·4	0	0·0				
Soda, diet	274	43·4	156	24·7	191	75·2	0	0·0	118	89·4	13	12·4	0	0·0				
Soda, unclear||	9	1·4	2	0·3	253	99·6	0	0·0	8	6·1	97	92·4	0	0·0				
Other pre-sweetened beverage¶	275	43·6	152	24·1	195	76·8	0	0·0	123	93·2	8	7·6	8	2·9	0·47	0·27–0·53	10·2	8·9–11·4
Other unsweetened/artificially sweetened beverage**	130	20·6	12	1·9	243	95·7	0	0·0	118	90·1	13	12·4	0	0·0				
Unspecified fountain/kid’s drink option††	71	11·3	49	7·8	231	90·9	4	0·6	22	16·8	83	79·1	2	2·8	0·10	0·10–0·10	2·3	2·1–2·6

Nine observations did not allow a beverage choice/selection with children’s meals sold online, but presumably could be selected at pick-up.

* For sampled restaurants (*n* 254), there were four potential ordering platforms sampled per restaurant (restaurant specific platform; DoorDash, GrubHub and UberEats), resulting in a potential four observations/restaurant.

† Includes observations from the first screen where beverage selection was available when ordering a children’s meal online, and in some cases, from a second screen if there was an option on the first screen to see additional beverage choices.

‡ Thirty-six observations did not have an image of the children’s meal that showed a beverage on any of the observed ordering screens.

§ California Senate Bill (SB) 1192, Healthy-by-Default Children’s Meal Beverage Law, requires restaurants that serve a children’s meal which includes a beverage make the default beverage offered with the children’s meal to be one or more of the following: 1. Water, sparkling water, or flavoured water with no added natural or artificial sweeteners; 2. Unflavoured milk (plain dairy milk); 3. Non-dairy milk alternative (example: almond, coconut or soy milk). It does not prohibit a restaurant’s ability to sell, or a customer’s ability to purchase, an alternative beverage instead of the default beverage offered with the children’s meals, if requested by the purchaser of the children’s meal.

|| Was not clear by online listing if beverage was flavoured or unflavoured milk or regular or diet soda.

¶ Other pre-sweetened beverages included sweetened iced teas, sweetened lemonades, sweetened juice drinks and milkshakes.

** Other artificially or unsweetened beverages included almost exclusively unsweetened iced teas (98 %), but also included lite lemonade (2 %).

†† Includes items listed as ‘fountain drink,’ ‘kid’s drink,’ ‘small drink,’ or something similarly non-specific, often with a link or drop-down menu with specific beverage options, including sugar-sweetened beverages.

Only 132 observations (20·9 %) had beverages available on both a first and second screen. The most common beverages offered on the second screen were other unsweetened/artificially sweetened beverages (such as iced tea; 93·2 %), other pre-sweetened beverages (such as lemonade; 92·4 %), regular soda (89·4 %) and diet soda (89·4 %). Water and unflavoured milk were offered on the second screen for 65·2 % and 59·1 % of observations, respectively. Water was offered consistently on the second screen across all platforms for 19·1 % of QSR sites; 26·7 % of QSR sites offered unflavoured milk consistently on the second screen across all platforms.

Among all beverages, water was the most likely to have an additional cost (upcharge); among the 445 observations that offered water, 41·1 % had a water upcharge. Among the 396 observations that offered unflavoured milk, 10·6 % had an upcharge. Of the 275 observations that offered other presweetened beverages 2·9 % had an upcharge for at least one of those drinks. Of the 71 observations that offered an unspecified kids’/fountain drink option, only 2·8 % upcharged for at least one of those drinks. Juice was also frequently upcharged; of the 326 observations that included 100 % juice, 92 (28·4 %) had an upcharge and of the 166 that offered diluted juice 13 (7·8 %) had an upcharge. No observations upcharged for soda (regular, diet or unclear), or other unsweetened/artificially sweetened beverages. The average upcharge cost for water was $0·51, which on average represented an 11 % increase over the total children’s meal cost. On average, 100 % fruit juice had the most expensive upcharge (average of $1·03, representing 24 % of the average total children’s meal cost).

Using the most liberal criteria (Criteria 1), less than half of the observations (38·5 % of restaurant-specific platforms and 41·7 % of third-party platform observations; 40·5 % of all platform observations combined) implemented SB1192 (Fig. 2). If we also consider an upcharge for the allowed default beverages to be inconsistent with SB1192 (Criteria 2), then implementation rates drop considerably to only 14·0 % for restaurant-specific platform observations, 8·8 % for third-party platform observations (10·7 % for all platform observations combined). Disallowing an unspecified kids’/fountain drink option on the first beverage ordering screen (Criteria 3) lowered implementation consistency only slightly compared with Criteria 2. No observed restaurant-specific platform observations and only 8·8 % of the third-party platform observations (5·6 % of observations from all platforms combined) met the most stringent criteria (Criteria 4; only the allowed default beverages were offered on the first beverage ordering screen, with no links to additional options and no upcharges for allowable default beverages).

## Discussion

Based on observations from restaurant websites and three of the most popular online ordering services, most California QSR located in low-income neighbourhoods are not offering children’s meals that are consistent with SB1192, the state’s Healthy-By-Default Children’s Meal Beverage law, thereby diminishing the potential impacts of the legislation in reducing SSB intake among children. Further, additional costs for healthy default beverages (water and unflavoured milk) may also be discouraging families from choosing those beverages with children’s meals. Together, low consistency with SB1192 and more prevalent upcharges for default beverages, coupled with no upcharges for soda or fountain drinks (which often contain sugar), could be mitigating progress that has been made with regards to SB1192 adherence in physical QSR spaces to improve children’s diets.

Prior work examining California QSR beverage offerings with children’s meals before and after implementation of SB1192 demonstrated that the number of in-restaurant and drive-through QSR menus including only law-consistent beverages significantly increased nearly sixfold, from 10 % to 66 %^(34)^. However, 1-year post SB1192 implementation, one-third of sampled QSR were still not consistent with the law in regard to the menu board and only 1 % were offering beverages during in-person ordering in a manner consistent with the legislation. Interestingly, the same study found offerings on menus/menu boards did not change after similar legislation was passed in Wilmington, Delaware. This could be because no restaurant managers in Wilmington reported knowing about the law, compared with nearly one-third of California managers; or, it could be because a smaller proportion of Wilmington restaurants were chains and chains might be expected to have increased awareness and more systematic implementation of applicable legislation^(34)^. Online ordering platforms, however, were not examined and to our knowledge there is no published literature on the implementation of SB1192 or similar legislation on online ordering platforms.

The text of SB1192 lacks clear and specific language in several regards. First, it does not mention online ordering platforms, a concerning omission given the rise in online and on-site kiosk ordering in restaurants, which accelerated during COVID-19 restrictions beginning in March 2020. Second, no specific reference is made to upcharges for default beverages. And third, it is not clear how online platforms could present only the allowable defaults beverages while also allowing customers to request other beverage options. Given this lack of clarity in legislative language, we examined several increasingly restrictive criteria for implementation consistency with SB1192. Implementation was low (under 41 %) regardless of the criteria used, and fewer than 6 % of observations had only water or unflavoured milk available on the first online ordering screen where beverages can be chosen, with no upcharge for those beverages.

In this study, while water, unflavoured milk and 100 % fruit juice were the most frequently offered beverages on the first online ordering screen, these beverages were also the most likely to be available only at an additional cost. When there was an upcharge, choosing water with a children’s meal increased the cost of the meal by 11 %; for unflavoured milk, by 7 %. In contrast, no observations upcharged for soda (regular and diet) or fountain drinks, and very few (< 3 %) upcharged for other pre-sweetened beverages.

Upcharges for the allowable default beverages are clearly contrary to the intent of SB1192 because the upcharges likely discourage those selections among price-conscious consumers. Prior evidence shows that pricing impacts beverage choice^(30)^, with studies conducted in stores demonstrating that cost, in combination with promotion and prompting, effectively impacts purchasing behaviour^(31)^. Price is an especially important factor for low-income consumers, who are significantly more conscious of cost and value than higher income consumers^(38)^. Higher costs for healthy default beverages sold online from QSR in low-income, majority Latinx neighbourhoods, coupled with no additional cost for SSB such as soda and pre-sweetened drinks, likely discourages consumers from making healthier selections with children’s meals sold online. Yet, this study found that 41 % of observations that offered water had an upcharge for water, and 10 % that offered unflavoured milk, and 20 % that offered an unspecified milk option, upcharged for those beverages. These upcharges not only undermine the intent of SB1192, but also likely contribute to persistent disparities in SSB consumption between lower income and higher income youth and among children of colour compared with non-Hispanic White children^(39)^.

These findings suggest that restaurants were unaware of the legislation or uncertain as to whether, and how, to apply the mandate to online ordering; 59·5 % of the observations included options that were clearly not compliant such as SSB, artificially sweetened beverages or unsweetened tea (Fig. 2, Criteria 1). Some (nearly 8 %) had the option to choose a ‘fountain’, ‘kids’, or ‘small drink’ that often included a drop-down or link to a list that included options other than the allowable default beverages. In many instances, there was an equally prominently displayed option to click on a link to see ‘more beverages.’ This link sometimes included a photo of beverages that were clearly neither water nor unsweetened milk. One could argue whether listing these more generic options in this manner is consistent with the legislation. However, the effort involved in one click may not be a sufficient deterrent to selecting unhealthy options. Including a more inconspicuous link without photos or suggestive language as to the nature of other beverage options would be more consistent with the intent of this law.

These findings suggest the need to provide clarification to, and education for, those responsible for implementing SB1192 at QSR. Local and state agencies and their partners could work with restaurants and online ordering platforms to ensure complete implementation in a way that is most likely to reduce youth SSB consumption (the intent of the law). Changes at the restaurant chain or online platform level could impact not only the QSR and patrons in this sample, but restaurants and customers across the state. Otherwise, the opportunity presented by this legislation to influence the choices and preferences of young children will likely go largely unrealised. For those contemplating similar legislation, our findings suggest that they would be well-advised to include more specific language regarding online ordering and explicitly prohibit upcharges for allowable default beverages. No legislation can ever fully anticipate new developments or cover all possible scenarios in detail. Therefore, work by local authorities and community partners may be needed to ensure policies are fully implemented to maximise the benefit for the populations they are meant to protect and to reduce disparities.

Several study limitations necessitate mention. First, SB1192 does not contain clear language pursuant to beverages included with children’s meals sold online, making adherence to the law subject to interpretation. Second, we do not have data on QSR’ online children’s meals offerings prior to implementation of SB1192, precluding a pre-/post-examination of change. Collecting this data again, as online ordering and ordering by scanning Quick Response (QR) codes on personal devices inside of restaurants continues to increase in popularity, would provide additional important evidence on changes in consistency with SB1192 implementation over time. Third, we were not able to examine trends in consumer purchasing, nor study whether consistency with SB1192 impacted QSR’ bottom lines. However, evidence suggests that healthy default beverages are acceptable to both parents and children and do not decrease sales^(33,40–48)^. In fact, offering healthier options at QSR does not negatively affect corporate performance^(40,46)^ and may even have positive financial impacts^(29,49)^. Finally, our study sample includes QSR in low-income California neighbourhoods that were able to stay open during the COVID-19 pandemic, which could impact generalisability of these findings to QSR in higher income neighbourhoods or in other states.

For any legislation to have the intended effect, it is necessary for the legislation to be implemented. The intent of SB1192 is to reduce the consumption of SSB among young children^(21)^ by making healthy beverage choices (i.e. unsweetened water and milk or milk alternatives) the easy (i.e. default) option when ordering a restaurant children’s meal. The strength of this behavioural economics approach is that consumer education is not necessary to influence behaviour because conscious effort on the part of the consumer is not involved^(50)^. In addition, the purchaser must exert effort to select an unhealthy beverage option^(50)^. On average, QSR located in low-income California neighbourhoods are not offering beverages with children’s meals sold online in a way that’s consistent with the state’s Healthy-By-Default Children’s Meal Beverage law, thereby diminishing the potential impacts of the legislation in reducing SSB intake among children. As web-based ordering, ordering at mobile kiosks and ordering by scanning QR codes on personal devices inside of restaurants (rather than with a person at the counter) become increasingly common, further work to ensure QSR are offering healthy default beverages with children’s meals sold online is warranted.
